# Research on driving mechanism and prediction of electric power carbon emission in Gansu Province under dual-carbon target

**DOI:** 10.1038/s41598-024-55721-2

**Published:** 2024-03-13

**Authors:** Fuwei Qiao, Qinzhe Yang, Wei Shi, Xuedi Yang, Guanwen Ouyang, Lulu Zhao

**Affiliations:** 1 College of Economics, Northwest Normal University, Lanzhou, 730070 China; 2 College of Geography and Environmental Sciences, Northwest Normal University, Lanzhou, 730070 China; 3 College of Resources and Environment, Lanzhou University, Lanzhou, 730070 China

**Keywords:** Power industry, Carbon emissions, Ridge regression, Scenario prediction, Environmental sciences, Environmental social sciences

## Abstract

The electric power industry is a key industry for the country to achieve the double carbon target. Its low carbon development has a double effect on this industry and helps other industries to achieve the carbon peak target. This paper firstly uses the IPCC inventory method to calculate carbon emissions in the production phase of the power industry in Gansu Province from 2000 to 2019, followed by the ridge regression method and the STIRPAT model to analyse the quantitative impact of six major drivers on carbon emissions, and finally, the scenario analysis method is used to forecast carbon emissions in this phase. The results show that the carbon emissions of Gansu Province show a trend of rising and then falling, and reached a peak of 65.66 million tons in 2013. For every 1% increase in population effect, urbanisation level, affluence, clean energy generation share, technology level and industrial structure, carbon emissions will grow by 4.939%, 0.625%, 0.224%, − 0.259%, 0.063% and 0.022% respectively. Because of the clean energy advantage in Gansu Province, the low-carbon development scenario will continue to have low carbon emissions during the scenario cycle, which can be reduced to 53.454 million tons in 2030; the baseline scenario will achieve a carbon peak in 2025, with a peak of 62.627 million tons; the economic development scenario has not achieved carbon peak during the scenario cycle, and carbon emissions will increase to 73.223 million tons in 2030.

## Introduction

Global warming is a huge environmental problem facing all mankind at present, and "low carbon, sustainable and high quality" has become an inevitable trend of economic development of all countries. The U.S. government has planned to reduce carbon emissions 50–52% below 2005 levels in 2030, and achieve carbon neutrality by 2050. The European Union also made clear the goal of achieving carbon neutrality by 2050 through legislation in March 2020. On September 22, 2020, China made a commitment at the United Nations General Assembly: "Strive to peak CO_2_ emissions before 2030, and achieve carbon neutrality before 2060". This commitment not only demonstrates our country's responsibility as a major country, but also reflects our determination to manage the environment. As the largest carbon emitter in the world, China faces a significant challenge in reducing its emissions^1^. The electric power industry, as the industry with the largest carbon emissions in China, accounts for more than 40% of the country's total emissions^2^. Therefore, the key to effectively reduce carbon emissions is to clarify the influencing factors of carbon emissions in the power sector and to forecast and regulate future carbon emissions^3,4^. As an important ecological barrier and energy base in northwest China, Gansu Province has a large area of rich solar radiation resources and a relatively rich area. Gansu Province has unique comprehensive advantages in the development of new energy. In recent years, the government has actively promoted the development of clean energy power generation and achieved positive results. By the end of 2022, Gansu's installed capacity of clean energy, including hydropower, will account for about 65%, which is much higher than the national average of 48.6%^5^. But at the same time, Gansu province has some practical problems, such as backward economic development and lower per capita GDP than most provinces. Therefore, it is of great significance for the future development of the power industry and environmental regulation of Gansu province to work out the most suitable development path while realizing the goal of "dual carbon" in the power industry.

## Literature review

With the rapid development of industrialization and urbanization in China, the energy efficiency of the electric power industry has also improved substantially. However, at present, the energy structure of China's power sector is still not ideal, and the production process relies too much on the combustion of fossil energy^6^, resulting in a large gap between the resource consumption and carbon emissions of the industry and the international advanced level. With the promotion of the "dual carbon" goal, electrification and the deep decarbonization of the power system have become an inevitable trend^7^. In recent years, many scholars have conducted a lot of research on electricity carbon emissions, and the relevant domestic and foreign researches related to this paper mainly include the following aspects: Accurate accounting of carbon emissions is the premise of analyzing the characteristics of carbon emissions and formulating emission reduction measures. At present, the international research methods on carbon emission accounting have been relatively mature, mainly focusing on emission factor method, mass balance method and measurement method^8^. Among them, the emission factor method is widely used by many scholars because of its advantages of convenient calculation and high applicability^9^. This method calculates carbon emissions with fuel consumption and carbon emission factors by fuel , and the emission factor values are often published by authoritative organizations, such as the 2006 IPCC Guidelines for National Greenhouse Gas Inventories issued by the United Nations Intergovernmental Panel on Climate Change (IPCC), and the Guidelines for the Preparation of Provincial-level Greenhouse Gas Inventories (for Trial Implementation) issued by China, etc.^10^; For example, Abdul-Wahab A et al.^11^ used the IPCC Guidelines to measure the carbon emissions generated by oil and natural gas consumption in the Sultanate of Oman, and found that its carbon emissions increased greatly with the increase in oil and natural gas consumption. Domestic scholar^12^ calculated China's interprovincial carbon emissions from 1995 to 2009 based on the IPCC greenhouse gas inventory, and further concluded that economic development, technological progress and industrial structure are the most important factors affecting China's carbon dioxide emissions. In addition, different scholars have calculated and studied carbon emissions from different perspectives of the power production process. Shi et al.^13^ calculated carbon emissions of the power system in Gansu Province from multiple perspectives including the production side, transmission side and consumption side, allowing the influence of power trade on regional power carbon emissions to be calculated and improving the fairness of regional emission reduction policy formulation.

To analyze the change of carbon emissions in a specific geographical range, to find out the drivers of carbon emissions and to identify the contribution of the drivers is conducive to more clearly identifying the problem and formulating targeted low-carbon policies. Economic growth, population size, urbanization level, power production structure, and energy intensity are generally considered to be the main influencing factors of electricity carbon emissions^10^. The decomposition methods of influencing factors on carbon emissions mainly include structural decomposition analysis (SDA) and indes decomposition analysis (IDA). The structural decomposition method includes many methods, and the main applications include EKC curve^14^, IPAT equation^15^ and STIRPAT model^16^. Among them, EKC curve is often used to investigate the inverse "U" shaped relationship between economic growth and environmental quality, while IPAT equation measures the impact of economic development, population effect and technological level on environmental quality. STIRPAT model is an extended form of IPAT equation, and is usually used by domestic and foreign scholars to empirically analyze the relationship between carbon emission and its influencing factors. Chen Zhanming et al.^17^ adopted the STIRPAT model to analyze the influencing factors of carbon emissions in cities above prefecture level in China, and found that population size, the proportion of the output value of the secondary industry and the growth of heating demand would significantly increase a city's carbon emissions. Shahbaz M^18^ used STIRPAT model to explore the impact of urbanization on carbon emissions in Malaysia, and found a U-shaped relationship between urbanization and carbon emissions. In addition, after analyzing carbon emission and its influencing factors, some scholars further used STIRPAT model and scenario setting method to predict future carbon emission^19–21^. Because STIRPAT model overcomes the drawback that all variables in IPAT equation affect carbon emissions in the same proportion, and can consider the impact of multiple factors on carbon emissions^22,23^, in this paper we selected STIRPAT model combined with scenario setting to study carbon emissions in the power production stage of Gansu Province.

To sum up, the issue of carbon emission reduction under the background of "dual carbon" has received much attention, and the research on the calculation and empirical analysis of carbon emissions has become increasingly mature. However, there are still problems such as inter-regional development imbalance in China, and there are large gaps in resource endowments and economic development stages among different regions. The realization of the dual carbon goal needs to be differentiated according to the specific conditions of different regions. And there are still few researches on a specific industry in less developed areas by now. At present, Gansu Province is still a region with high energy intensity, and the demand for sustainable development of ecological economy is particularly urgent^24^. In this paper, STIRPAT model is used to make an empirical analysis of the carbon emissions in the power production stage of Gansu Province, and different scenarios are set to predict the future carbon emissions, which is helpful to promote the low-carbon sustainable development of the power industry in Gansu Province, and has a forward-looking significance for environmental protection and the realization of regional dual carbon goals.

## Research methods and data sources

### Research technique

#### IPCC Carbon inventory preparation method

IPCC carbon inventory method is a relatively simple and practical carbon emission accounting method at present, which is widely used because of its strong applicability and low cost. Therefore, this paper adopts the carbon emission coefficient published by the United Nations Intergovernmental Panel on Climate Change (IPCC) to multiply the consumption of various fuels in the power production stage. The carbon emission of electricity production in Gansu Province is calculated^25,26^. The specific calculation formula is as follows:

1 $$CEP = \sum\limits_{k = 1}^{m} {ef_{k} } \times fm_{k} ,$$

where, $ef_{k}$ denotes the CO2 emission factor of the k fuel in the electric power production stage, and $fm_{k}$ denotes the consumption of the k fuel in the electric power production stage. The standard coal coefficients and CO2 emission factors of various fuels are shown in Table 1^13^.

**Table 1:** Caculation parametes of carbon emissions for different types of fossil fuel energy.

Types of energy	Raw coal	Coke	Crude oil	Gasoline	Kerosene	Diesel	Natural gas	Fuel oil
Discounted standard coal coefficient (10^4^tce/10^4^t)	0.7143	0.9714	1.4286	1.4714	1.4714	1.4571	1.3300	1.4286
Carbon emission coefficient (10^4^t/10^4^tce)	0.7559	0.8550	0.5857	0.5538	0.5714	0.5921	0.4483	0.6185

#### STIRPAT model

IPAT model uses population, affluence and scientific and technological level to measure environmental pressure comprehensively, but IPAT model has some limitations. In this model, each influencing factor can only change in the same proportion. York et al.^27^ modified the traditional IPAT model to obtain the random effects model (STIRPAT). The STIRPAT model can not only estimate each coefficient as a parameter, but also allow appropriate splitting of each influencing factor under the condition of theoretical consistency and need^28^ ,a large number of researches have improved this model to some extent according to their different research characteristics^16,18,29^. In order to study the carbon emission in the power production stage of Gansu Province, combined with the specific situation of Gansu Province, the STIRPAT model is extended as follows:

2 $$\ln I = \ln a + b\ln A + c\ln P + d\ln T + f\ln U + g\ln IS + h\ln C,$$

where: I represents the total carbon emission in the power production stage of Gansu Province (t); A represents affluence, expressed by the per capita GDP of Gansu Province over the years (yuan/person); P is the population effect, expressed by the number of people aged 15–65 in Gansu Province (10,000); T is the level of science and technology, expressed by the number of patents granted; U is the level of urbanization, expressed by the ratio of urban population to permanent population; IS is the industrial structure, expressed by the proportion of the added value of the secondary industry in GDP; C is energy cleanliness, expressed by the proportion of clean energy generation in total power generation; a is the model coefficient, and b, c, d, f, g, h are the elastic coefficients.

### Scenario setting

The change rate of each explanatory variable in Eq. (2) was set according to the regional development and policy planning^21,30^, and the predicted value of Gansu's electric power carbon emission was obtained by substituting them into the STIRPAT model, so as to provide a reference for achieving the carbon peak target in Gansu Province. In this study, all parameter changes were set as high growth rate, medium growth rate and low growth rate. The scenario cycle was set in 2020–2030, with 2020–2025 as the first phase and 2026–2030 as the second phase. In addition, three forecasting scenarios, low-carbon scenario, benchmark scenario and economic development scenario, are set. The change speed of each parameter in the three scenarios is shown in Table 2.

**Table 2:** Scenario setting of electric power carbon emission forecast in Gansu Province.

Explanatory variable	Low carbon scenario	Base scenario	Economic development scenario
Population effect	Low	Medium	High
Affluence	Low	Medium	High
Scientific and technological level	Low	Medium	High
Urbanization level	Low	Medium	High
Industrial structure	High	Medium	Low
Energy cleanliness	High	Medium	Low

#### Population effect

This paper selects the labor force population to reflect the population effect. The reason is that Gansu Province has a large population flow in recent years, and the selection of labor population can better highlight the impact of permanent population on carbon emissions. From 2000 to 2019, the population of Gansu Province at the end of each year showed a trend of first increasing and then decreasing, and reached a peak in 2010, followed by an average annual growth rate of − 0.22%. According to the National Population Development Plan (2016–2030), the inertia of growth in the total population size of the country is weakening from the current to 2030, and the working-age population will be decreasing at a faster rate during the period of 2021–2030. Therefore, the high, medium and low growth scenarios of the population effect growth rate in the first and second stages are set as − 0.12%, − 0.22%, − 0.32% and − 0.35%, − 0.45%, − 0.55%, respectively.

#### Affluence

In this paper, per capita GDP of a region is selected to represent the richness of the region. In accordance with the Outline of the 14th Five-Year Plan and Long-term Goals for 2035 issued by the People's Government of Gansu Province (hereinafter referred to as the Outline), the GDP of Gansu Province is set to grow at an average annual rate of 6.5% in 2021–2025; Considering the reasons that the economic growth rate will gradually decrease with the improvement of regional development level, the high, medium and low growth scenarios of the first and second stages of prosperity growth rate are set at 8.0%, 6.5% and 5.0% and 6.5%, 5% and 3.5% respectively.

#### Technology level

This paper selects the number of regional patent grants to measure the level of science and technology. According to the Science and Technology Innovation Plan of Gansu Province in the Outline, the number of high-value invention patents per 10,000 people in Gansu Province will increase from 0.986 to 1.32 in 2020–2025, with a total increase of 33.87% and an average annual growth rate of 6.0%; By analogy with the data growth rate, this study sets the high, medium and low growth scenarios of the average annual growth rate of the first and second stages of science and technology level as 7.0%, 6.0%, 5.0% and 6.0%, 5.0% and 4.0% respectively.

#### Urbanization level

In this paper, the urbanization rate reflects the regional urbanization level. According to the plan in the Outline, the urbanization rate of Gansu Province will reach 58% in 2025, with an average annual growth rate of 2.3%; At present, the national urbanization rate is 64.72%, in contrast, the urbanization rate of Gansu Province still has a relatively high room for growth, and can still maintain the planned urbanization rate growth rate in the future. Therefore, the high, medium and low growth scenarios of the growth rate of the first and second stage urbanization levels are set at 3%, 2.3% and 1.6%.

#### Industrial structure

This paper selects the proportion of added value of secondary industry in GDP to characterize industrial structure. By calculating the change rate of the proportion of secondary industry in the last 5 years, we concluded that the average annual growth rate is − 3.7%. Therefore, the high, medium and low growth scenarios of the proportion of secondary industry in the first and second stages are set as − 5.0%, − 3.7%, − 2.4% and − 4.3%, − 3%, − 1.7% respectively.

#### Energy cleanliness

In this paper, the proportion of clean energy generation in total power generation reflects energy cleanliness. According to the instructions in the "14th Five-Year Plan for Energy Development in Gansu Province", the proportion of clean energy generation in Gansu Province will reach 71.94% in 2025, with an average annual growth rate of 4.1%. The construction of clean energy power generation is more vulnerable to environmental, weather and other factors, so thermal power is difficult to be completely replaced in a short time. Therefore, the high, medium and low growth scenarios of the growth rate of clean energy power generation in Gansu Province in the first and second cycles are set as 4.5%, 4.1%, 3.7% and 2.4%, 2.0%, 1.6%, respectively.

### Data sources

In the calculation of direct CO2 emissions in this study, the fuel consumption data were all derived from the energy balance sheet of Gansu Province in the Energy Yearbook, and the carbon emission coefficients of various fossil energy sources were derived from the 2006 IPCC Guidelines for National Greenhouse Gas Inventories issued by the Intergovernmental Panel on Climate Change (IPCC). The data of total population, population age structure, per capita GDP, gross industrial added value, urbanization rate, and number of patents granted in Gansu Province are all from China Statistical Yearbook and Gansu Development Yearbook from 2000 to 2019, and the data of power generation from different energy sources are from the Statistical Data Collection of Electric Power.

## Interpretation of result

### Analysis of carbon emission characteristics of electricity production

**Figure 1:**
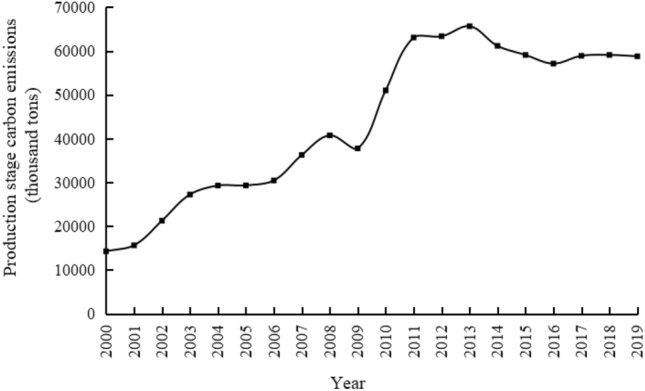
Carbon emissions during the power production stage in Gansu Province from 2000 to 2019.

During the study period, carbon emissions in the power production stage of Gansu Province showed a trend of first fluctuating, rising, reaching a peak, then decreasing and then slightly fluctuating (Fig. 1). From 2000 to 2008, there was an overall upward trend, from 14,254 kilotons in 2000 to 40,737 kilotons in 2008, with an average annual growth rate of 14.0%; This was subsequently reduced to 37,753 kilotons in 2009; From 2009 to 2013, the carbon emissions in the power production stage showed a rapid first and then a gentle upward trend, and reached a peak of 65,664 kilotons in 2013. The average annual growth rate during this period was 14.8%. After 2013, with China's largest onshore 3 MW fan demonstration base officially completed in Yumen City, Guazhou County 100 MW photovoltaic power generation project successfully connected to the grid, Gansu Province photovoltaic, wind power installed capacity reached 4.2984 million kilowatts and 7.0281 million kilowatts, respectively, ranked first and third in the country at that time; The significant increase in the proportion of clean energy generation such as wind power generation in Gansu Province, as well as the decline in the carbon emission intensity of power production to a certain extent caused by technological progress, the carbon emission of electricity in Gansu Province showed a significant downward trend from 2013 to 2016, with an average annual decline rate of 4.5%. The period from 2016 to 2019 showed a small fluctuation, with an average rate of change of 1.32%.

### Empirical analysis based on the extended STIRTAR model

In order to test the interpretation effect of independent variables on dependent variables, the least square method was first used to perform multiple linear regression analysis on each data. The results showed that most variables could not reject the null hypothesis at the significance level of 10%, and the VIF value of the remaining explanatory variables was greater than 10 except the proportion of labor population and clean energy power generation. The VIF values of affluence and urbanization level are greater than 100, 251.009 and 313.768, respectively, indicating that there is serious multicollinearity among the variables in this model. Ridge regression, as an improved least squares estimation method, has the characteristic of getting more realistic and reliable regression coefficient at the cost of losing part of information and accuracy. It is often used for regression analysis of collinear data to eliminate the influence of multicollinearity.In this paper, referring to the practice of Huang Rui et al.^31^, ridge regression method is adopted to eliminate the influence of multicollinearity.

**Figure 2:**
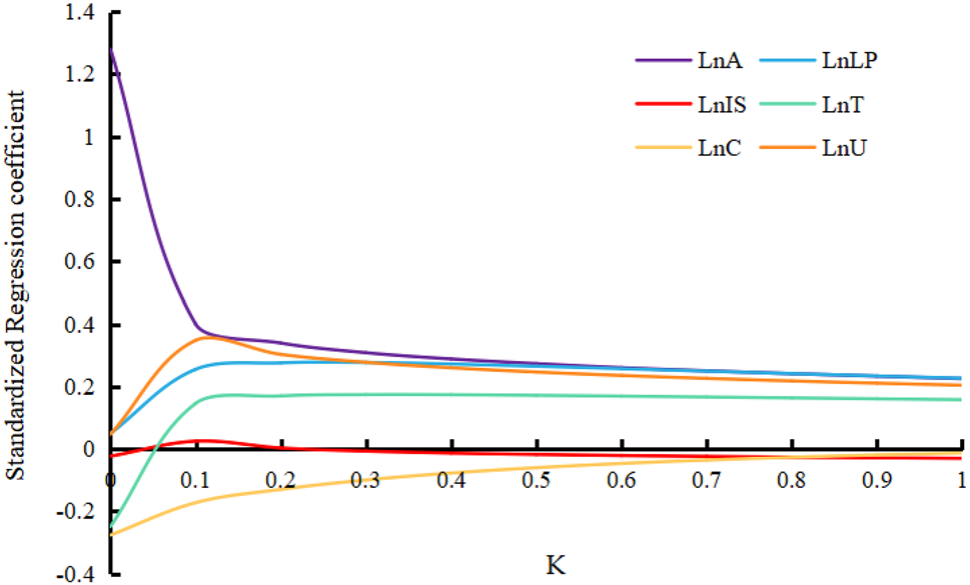
Ridge trace map.

The ridge trace map obtained by SPSS is shown in Fig. 2. The ridge parameters were selected from the ridge trace map. Generally, the smaller the ridge parameters are, the higher the goodness of fit of the equation will be. In this model, the optimal ridge parameter k = 0.18 was selected, and the ridge regression results obtained at this time are shown in Table 3.

**Table 3:** Results of ridge regression.

K = 0.18	B	Standard error	t	P	R^2^	F
Constant	− 23.547	5.904	− 3.988	0.002***	0.966	61.022 (0.000***)
lnP	4.939	0.802	6.159	0.000***		
lnA	0.224	0.019	11.778	0.000***		
lnT	0.063	0.013	4.857	0.000***		
lnU	0.625	0.056	11.078	0.000***		
lnC	− 0.259	0.098	− 2.63	0.021**		
lnIS	0.022	0.186	0.12	0.906		

** and *** indicate that statistics are significant at the 5%, and 1% level of significance, respectively.

As can be seen from Table 3, constant terms, lnA, lnP, lnT and lnU all passed the t test with a significance level of 1%, lnC passed the t test with a significance level of 5%, and the model as a whole passed the joint F test with a significance level of 1%, indicating that there is a good regression relationship between independent variables and dependent variables. The goodness of fit R^2^of the model is 0.97, indicating that the equation fits well. According to the coefficient of each explanatory variable, The factors can be ranked in descending order by their impact on carbon emissions as follows: Population effect > urbanization level > affluence > proportion of clean energy power generation > Science and technology level > industrial structure.where carbon emissions will increase by 4.939% when population effect increases by 1%, with other variables remaining unchanged,while Carbon emissions will increase by 0.625%, 0.224%, − 0.259%, 0.063% and 0.022%, respectively when urbanization level, affluence, proportion of clean energy power generation, science and technology level and industrial structure increase by 1%. According to the regression results, the direct impact of clean energy power generation on carbon emissions is not significant among the many influencing factors, but it does not mean that the positive impact of clean energy on carbon emissions at different stages of development is also limited. Relevant studies have pointed out that the increase in installed capacity of clean energy power generation will continue to reduce carbon emissions in different stages of development^32^. The calculated STIRPAT model is as follows:

3 $$\ln I = - 23.547 + 4.939\ln P + 0.224\ln A + 0.063\ln T + 0.625\ln U - 0.259\ln C + 0.022\ln IS.$$

**Figure 3:**
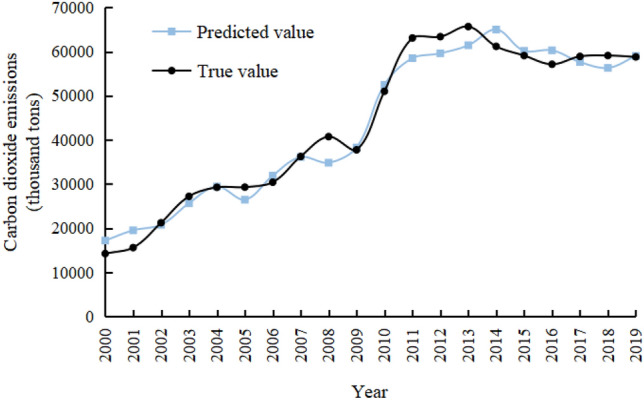
Comparison of the predicted and true carbon emissions from 2000 to 2019 in Gansu Province.

In order to ensure that this model can predict future carbon emissions more accurately, various data from 2000 to 2019 are substituted into model (3) to get the predicted value of carbon emissions. The calculated error between the predicted value and the actual value is small, with an average error rate of 6.4%, indicating that this model can be used to predict the future carbon emissions of Gansu Province. The comparison results are shown in Fig. 3.

### Future carbon emission prediction of Gansu Province

**Figure 4:**
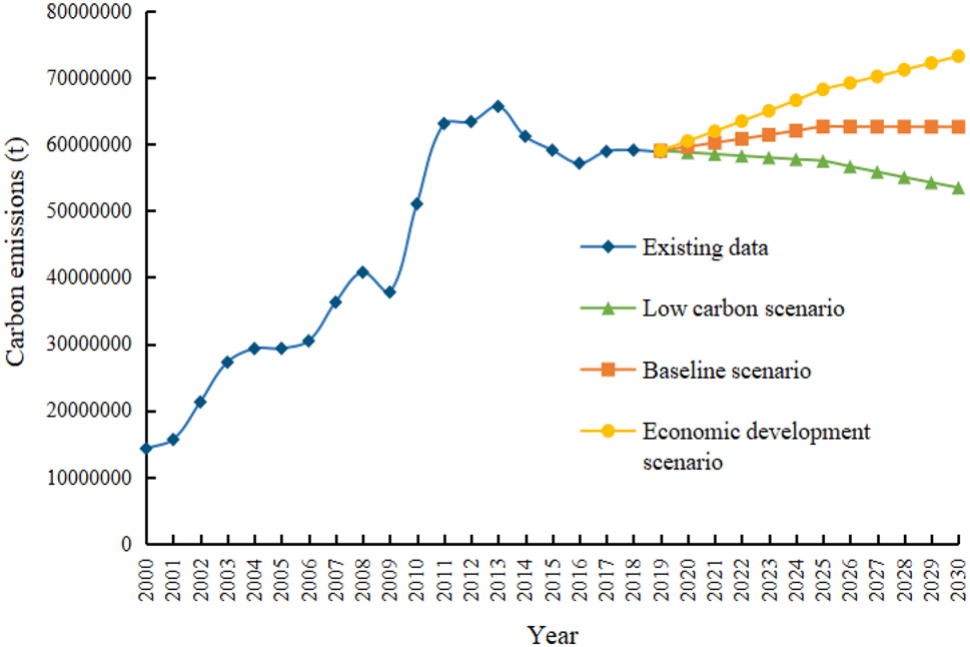
Prediction of carbon emission value of power production stage in Gansu Province under different scenarios.

Combining the above scenario settings, the future carbon emissions in the power production stage of Gansu Province are predicted based on the STIRPAT model, and the prediction results are shown in Fig. 4.

In the low-carbon scenario, carbon emissions show a continuous downward trend from 2020 to 2030. Combined with previous emission data, it can be seen that carbon emissions start to decline steadily from 2018, that is, the carbon in the power production stage reaches its peak in 2018^33^, with a peak value of 59.105 million tons. In the baseline scenario, carbon emissions show a trend of first rising and then declining, and reach the peak in 2025, which is 7 years later than that in the low-carbon scenario, with a peak of 62.627 million tons. Under the economic development scenario, carbon emissions show a continuous upward trend without a peak, and are predicted to reach 73.223 million tons in 2030.

As a major energy province and a golden location for the Belt and Road construction, Gansu Province should take economic construction as the focus of development while realizing environmental regulation. In this context, combined with the predicted results of this paper, the carbon peak in power production stage can be achieved in 2025 under the baseline scenario, which is in line with and ahead of China's carbon peak target. Economic growth and urban development also meet the actual development needs of Gansu Province. Therefore, the base scenario is the best scenario for the carbon peak development of power production in Gansu Province.

## Conclusion and suggestion

### Research conclusions

This paper firstly calculates the carbon emissions in the power production stage of Gansu Province by using the emission factor method and the carbon emission coefficient published by IPCC. Then, STIRPAT model is adopted to analyze the influence of 6 factors such as population effect, affluence, science and technology level, urbanization level, industrial structure and energy cleanliness on the carbon emissions in the power production stage of Gansu Province. In addition, three different scenarios are set to predict the carbon emissions in the power production stage of Gansu Province from 2020 to 2030, and the conclusions are as follows:

1. From 2000 to 2013, the carbon emissions in the power production stage of Gansu Province showed a fluctuating upward trend. After reaching the peak in 2013, the carbon emissions in the power production stage of Gansu Province showed a trend of slight fluctuation after a significant decline, and did not show a continuous upward trend.
2. At the present stage, population effect, affluence, science and technology level, urbanization level and industrial structure all play a positive role in promoting carbon emissions in the power production stage of Gansu Province, while energy cleanliness plays a reverse role in promoting carbon emissions; from the perspective of elasticity coefficient, population effect has the greatest influence on carbon emission.
3. Under the three different forecast scenarios, the low-carbon scenario and the base scenario reach the peak in 2018 and 2025 respectively, while the economic development scenario fails to reach the peak in the forecast period. The magnitude of the peak carbon emission when the carbon reaches the peak is: base scenario > Low carbon scenario. Based on the provincial situation and development status of Gansu province, this paper holds that the base scenario is the best development scenario of Gansu Province.

### Policy suggestion

Based on the above conclusions, the following suggestions are put forward: (1) Population effect is the most significant factor affecting carbon emissions in the stage of power production. Gansu Province should start with improving population quality and advocate the transformation of people's life to green and low-carbon life. The government can properly control relevant electricity prices to cultivate residents' awareness of energy conservation and increase the popularization of knowledge related to electricity consumption, so as to reduce electricity and energy consumption and speed up the process of carbon peaking. (2) As the urbanization rate is the second largest factor affecting carbon emissions in the power production stage, Gansu Province should continue to optimize the structure of urban and rural areas, take into account the construction of ecological civilization on the road of promoting urbanization, accelerate the low-carbon transformation of production and life in urban areas, constantly deepen the requirements of rural revitalization and development, and complete the emission reduction task with specific targets for different regions. (3) Affluence is the third major factor affecting carbon emissions in the power production stage of Gansu Province. At present, various economic development indicators of Gansu Province are still backward compared with most provinces in China, and the pressure of economic development and environmental protection coexist. Therefore, Gansu Province should make every effort to realize the planned economic development speed, strengthen economic and urban and rural construction, and give consideration to environmental protection. To achieve balanced development between economy and green ecology. (4) Adhere to the vigorous development of photovoltaic, hydropower, electricity and other clean energy power generation. Located in northwest China, Gansu Province is rich in wind energy and light energy resources. The increase in installed capacity of clean energy power generation can have a positive impact on regional CO_2_ emission reduction in the long run. Gansu Province should continue to improve the technology and quantity of clean energy power generation, and improve the efficiency of thermal power generation, so as to maximize its unique geographical advantages. (5) At present, the development level of clean energy power generation in Gansu Province is in a leading position in the country, and the proportion of clean energy power generation in 2019 reached 56.2%, while the proportion of clean energy power generation in China as a whole was 30.4%; combined with this research, It can be concluded that under the current planning and setting of development, the power sector of Gansu Province can still achieve peak carbon by 2025, 5 years earlier than the committed year of China’s peak carbon. However, the economic development of Gansu Province is in a backward position in the whole country, and the level of science and technology, resource elements and financial investment still cannot meet the needs of development. To sum up, within a certain range, Gansu Province can trade carbon emission rights with provinces or cities with large carbon emission and carbon emission intensity in exchange for resource factors and financial support, which will not only relieves the pressure of carbon emission reduction in other provinces, but also enable Gansu Province to obtain economic resource endowments, helping Gansu province to improve its development speed and quality under the new normal of economic development, narrow the gap between the scientific and technological level and the eastern coastal provinces, and achieve win–win results with the trading provinces.

### Research limitations

This study calculated the carbon emissions in the power production stage of Gansu Province, and further discussed its influencing factors and mechanism, providing suggestions and references for Gansu Province to balance economic development and ecological civilization construction in the future. However, there are still some limitations in this study:

1. Limited by the availability of data, the comprehensiveness of the selection of influencing factors for carbon emissions in the power production stage of Gansu Province may be affected by this study. This study fully considers the economic and human development characteristics of Gansu Province, but some relevant influencing factors may still be omitted.
2. About scenario setting, based on the development status of Gansu Province and national and regional development planning, this study accurately sets the future development of each influencing factor as much as possible; However, it has not been determined whether the actual development meets the expected planning, and this error will affect the accuracy of the predicted carbon emission value.

Based on the above, future research can further improve the span and breadth of data, screen and study the influencing factors of carbon emissions from an interdisciplinary perspective, so as to fully understand the development status and future development goals of the power production stage in Gansu Province, accurately promote the construction of ecological civilization, and encourage technological innovation and progress in related industries. Through the improvement of relevant research to accelerate the realization of China's dual carbon goal, to provide a reference for regions with similar development characteristics.

## Data Availability

The datasets used and analyzed during the current study are available from the corresponding author on reasonable request.
